# Grape seed powder improves renal failure of chronic kidney disease patients

**DOI:** 10.17179/excli2016-363

**Published:** 2016-06-27

**Authors:** Khaoula Turki, Kamel Charradi, Habib Boukhalfa, Monia Belhaj, Ferid Limam, Ezzedine Aouani

**Affiliations:** 1 University of Carthage, Faculty of Sciences of Bizerte, 7021 Jarzouna, Tunisia; 2 Laboratory of Bioactive Substances, Center of Biotechnology of Borj Cedria, BP 901, 2050 Hammam Lif, Tunisia; 3 Hemodialysis Unit, Regional Hospital of Menzel Bourguiba, Tunisia; 4 Hemodialysis Unit, Habib Bougatfa Hospital, Bizerte, Tunisia

**Keywords:** grape seed extract, oxidative stress, antioxidant status, chronic kidney disease, protection

## Abstract

Chronic kidney disease (CKD) is a syndrome characterized by progressive and irreversible deterioration of renal function linked to slow destruction of renal parenchyma, eventually terminating in death when sufficient number of nephrons are damaged. Oxidative stress is commonly observed in CKD patients resulting from an imbalance between overproduction of reactive oxygen species (ROS) and impairment of defence mechanisms. Grape seed extract (GSE) is a polyphenolic mixture exhibiting antioxidant and anti-inflammatory properties. We conducted an interventional pilot study of supplementation with GSE capsules (GSE group, n = 23) or placebo (control group, n = 10) on CKD patients. Blood and urine samples were collected at baseline and after a six-month-long supplementation period to determine some renal function biomarkers, as well as antioxidant, anti-inflammatory and haematological parameters. GSE improved glomerular filtration rate (GFR) and proteinuria, increased the anti-oxidant status as assessed by high plasma catalase and superoxide dismutase and also lowered lipoperoxidation and carbonylation. GSE ameliorated inflammation by decreasing CRP, triglyceridemia and counteracted anemia and thrombocytopenia. Supplementation with 2 g GSE/day for six months improved some kidney function parameters of CKD patients and this beneficial effect of GSE seems to be mediated at least partly by its antioxidant and anti-inflammatory properties.

## Introduction

Chronic kidney disease (CKD) is a slow and progressive decline of kidney function that is often linked to long standing diseases such as diabetes and hypertension (Remuzzi et al., 2002[35]). It is generally characterized by high amount of creatinine, low GFR and proteinuria which predict an accelerated loss of renal function that irreversibly leads to end stage renal disease (Ritz et al., 1999[36]). Oxidative stress and inflammation play important roles in both the progress of CKD and related complications (Massy and Nguyen-Khoa, 2002[30]). Oxidative stress results from an imbalance between ROS overproduction and reduced antioxidant defences within mitochondria, leading to its dysfunction and consequently to renal cell failure (Wallace, 2005[45]). 

Grape seed extract (GSE) is a complex polyphenolics mixture containing flavonoïds, non flavonoïds, proanthocyanidins exhibiting multi-organ protection in various experimental settings (Khanal et al., 2009[24]). For instance, GSE protects the heart (Charradi et al., 2011[11]), the liver (Charradi et al., 2014[8]), the brain (Charradi et al., 2012[10]) and the kidney (Charradi et al., 2013[9]) against high fat diet (HFD)-induced obesity and lipotoxicity in rat. Furthermore high dosage GSE was even shown to improve renal injury in type 2 diabetic rats through its anti-oxidant and anti-inflammatory properties (Bao et al., 2015[5]), and also to protect against arsenic (Zhang et al., 2014[47]), cisplatin (Gao et al., 2014[19]), amikacin (Ulusoy et al., 2012[43]), and cyclosporine A-induced nephrotoxicity (Ulusoy et al., 2012[44]).

The present work aims to test the ability of GSE to improve kidney dysfunction of CKD patients from various renal stages i.e stage 2, 3, 4. Data mainly show that although used at a low dosage, GSE improves several kidney function parameters as well as the anti-oxidative status of CKD patients after a 6-month-supplementation period.

## Material and Methods

### Subjects and study design

CKD patients with renal failure (stages 2, 3, 4) were recruited as volunteers at the haemodialysis unit of the Regional Hospital of Menzel Bourguiba for a randomized double blind and placebo control pilot study. All patients suffered from diabetic nephropathy and hypertension. They were treated with insulin (two doses per day), received captopril 50 mg (2 tablets/day) for hypertension, furosemide 40 mg (asylix 2 tablets/day) for nephropathy, folic acid (foldine 2 tablets/ day) and iron (2 tablets/day) for anemia. Key exclusion criteria included smokers and haemodialysis patients receiving antioxidant supplements in the previous six months. All patients were asked to keep their habits and medication as usual and were dispatched into two groups: experimental and placebo. Patients in the placebo group (n = 10) were distributed as follows:

- 3 patients were from stage 2 (GFR ≤ 90 ml/min/1.73m^2^)
- 4 patients were from stage 3 (GFR ≤ 60 ml/min/1.73m^2^)
- 3 patients were from stage 4 (GFR ≤ 30 ml/min/1.73m^2^)

Patients in the GSE group (n = 23) were distributed as follows:

- 6 patients were from stage 2
- 11 patients were from stage 3
- 6 patients were from stage 4

The GSE group was daily supplemented with six capsules of GSE, each capsule containing 350 mg of grape seed powder. Two bottles of 90 capsules each were provided to each patient for one month during the entire six months long study which lasted from May to October 2011. The placebo group received starch containing capsules in the same conditions. Blood samples were withdrawn after an overnight fasting at the beginning of the study and after 6 months of supplementation with either GSE or placebo. Urines were also collected at baseline and at the end of the experimentation for proteinuria determination. The study protocol was approved by the ethics committee on clinical research of the Regional Hospital of Menzel Bourguiba and informed consent was obtained for all participants. The procedures followed were in accordance with the Helsinki Declaration of 1975, as revised in 2000 and 2008.

### GSE preparation

GSE was processed from a grape cultivar (Carignan) of *Vitis vinifera* from northern Tunisia. Seeds were carefully separated from skin, washed, dried and grounded with an electric grounder until a fine powder was obtained. After mixing with 3 % colloïdal silica as excipient, the powder was conditioned into capsules weighting approximately 350 mg. Total polyphenolics content as well as polyphenol composition were determined previously (Charradi et al., 2014[8]).

### Biochemical analyses

Haematological parameters were determined on whole blood by using automated procedures. GFR was calculated by the Modification of the Diet Renal Disease (MDRD) method. Plasma was processed by centrifugation at 3000 rpm for 10 min and used for the determination of renal function biomarkers as urea and uric acid using commercial kits from Biomaghreb, Tunisia. Urine urea and uric acid were also determined and proteinuria evaluated using the biuret method (Ohnishi and Barr, 1978[34]). Oxidative stress was assessed by measuring plasma lipoperoxidation (Draper and Hadley, 1990[15]), protein carbonylation (Levine et al., 1990[26]), H_2_O_2_ (Kakinuma et al., 1979[23]), free iron (Leardi et al., 1998[25]) as well as by measuring antioxidant enzyme activities as catalase (CAT) (Aebi, 1974[1]), superoxide dismutase (SOD) (Misra and Fridovich, 1972[31]) and glutathione peroxidase (GPx) (Nakamura et al., 1974[33]).

Inflammation was evaluated using plasma CRP and LDH. Triglyceridemia and cholesterolemia were measured using commercial kits from Biomaghreb (Fossati and Prencipe, 1982[17]; Allain et al., 1974[2]) and plasma lipase using a previously described method (Humbert et al., 1997[21]).

### Statistical analysis 

Results were expressed as mean ± SEM. Data were compared by a non-parametric test (Kruskal-Wallis). P value less than 0.05 was considered signiﬁcant.

## Results

**Figure 1:**
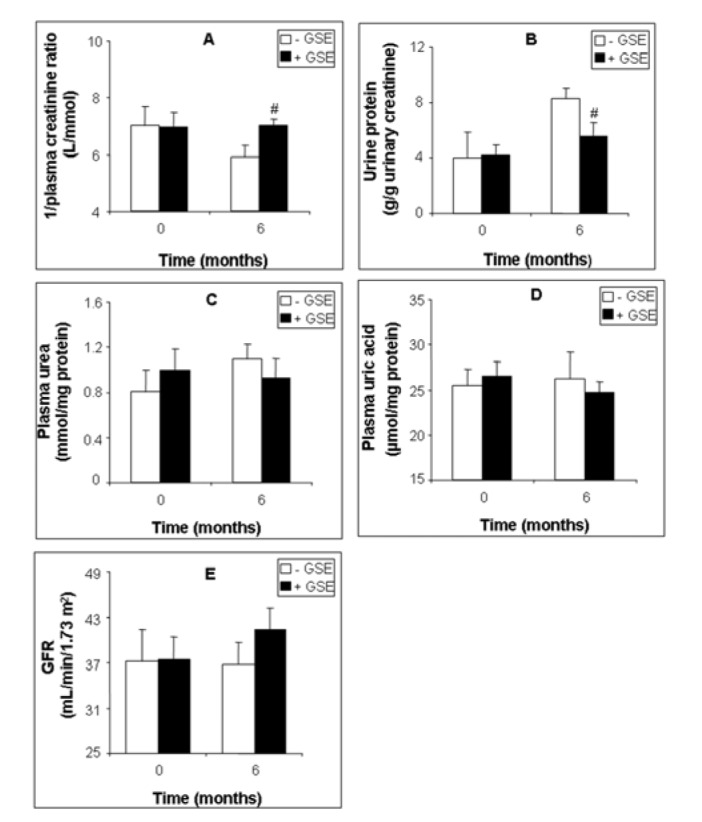
Figure 1: Effect of GSE supplementation on renal function biomarkers of CKD patients. 1/plasma creatinine ratio (A), proteinuria (B), plasma urea (C), plasma uric acid (D) and GFR (E). Data are presented as mean ± SEM. GSE (after 6 months treatment) vs. GSE (at 0 month): *p < 0.05. GSE (after 6 months treatment) vs. Placebo (after 6 months treatment): ^#^p < 0.05

We reported in Figure 1(Fig. 1) the effect of a 6-month-long supplementation with GSE on some renal hemodynamic parameters of patients suffering from kidney deficiency. GSE increased 1/plasma creatinine ratio by 19.42 % (Figure 1A(Fig. 1), p = 0.044), and decreased proteinuria by -32.67 % (Figure 1B(Fig. 1), p = 0.041) but had no significant effect on plasma urea (Figure 1C(Fig. 1)) and uric acid (Figure 1D(Fig. 1)). Moreover GSE slightly improved GFR by +18.70 % (36.74 ± 5.74 versus 43.62 ± 3.68, p = 0.604) (Figure 1E(Fig. 1)). 

**Figure 2:**
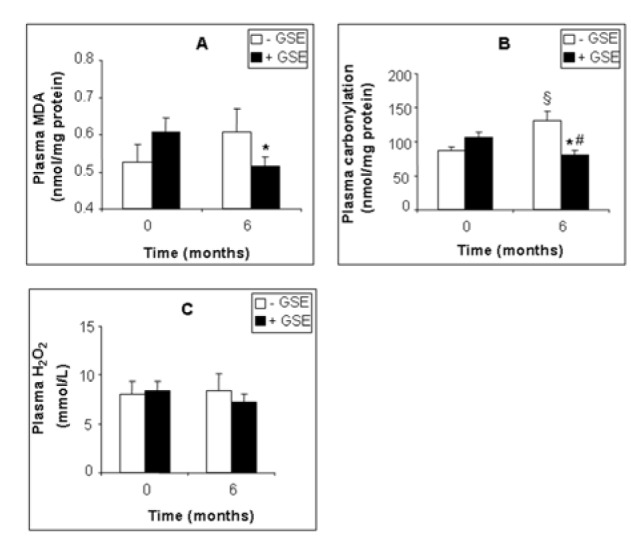
Effect of GSE supplementation on plasma pro-oxidant biomarkers of CKD patients. MDA (A), protein carbonylation (B), H_2_O_2 _(C). Data are presented as mean ±SEM. Placebo (after 6 months treatment) vs. Placebo (at 0 month): ^§^p < 0.05. GSE (after 6 months treatment) vs. GSE (at 0 month): *p < 0.05. GSE (after 6 months treatment) vs. Placebo (after 6 months treatment): ^#^p < 0.05

**Figure 3:**
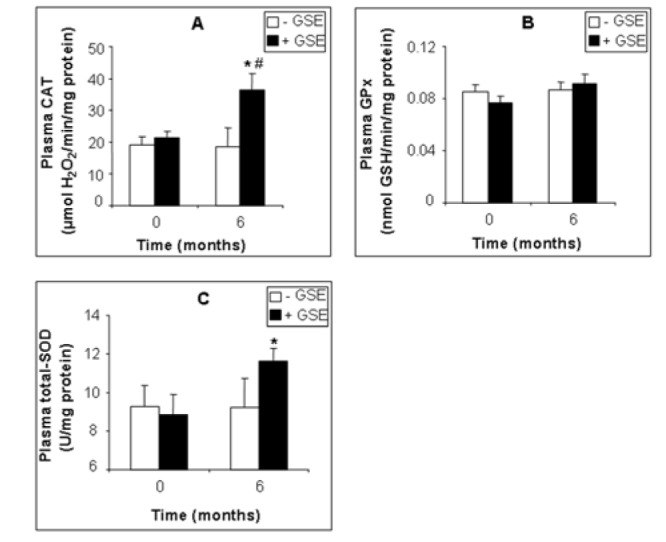
Effect of GSE supplementation on plasma antioxidant enzyme activities of CKD patients. CAT (A), GPx (B) and SOD (C) activity. Data are presented as mean ± SEM. GSE (after 6 months treatment) vs. GSE (at 0 month): *p < 0.05. GSE (after 6 months treatment) vs. Placebo (after 6 months treatment): ^#^p < 0.05

GSE also affected several plasma biomarkers of the antioxidant status. GSE slightly decreased plasma MDA by -14.96 % (Figure 2A(Fig. 2), p = 0.0307), and protein carbonylation by -38.82 % (Figure 2B(Fig. 2), p = 0.0319) but had no significant effect on hydrogen peroxide (-14 %, Figure 2C(Fig. 2), p = 0.383). Moreover GSE highly increased plasma CAT by +95.94 % (Figure 3A(Fig. 3), p = 0.015) and SOD by +25.99 % (Figure 3C(Fig. 3), p = 0.049) but had no effect on plasma GPx activity (Figure 3B(Fig. 3)). 

We reported in Table 1(Tab. 1) the effect of GSE on plasma biochemistry and inflammation of CKD patients. After a six-month-long treatment period, GSE partially alleviated plasma CRP by -12.81 % (+82.83 % for placebo, p = 0.028 against +56.40 % for GSE, p = 0.168), totally counteracted plasma LDH (p = 0.623) and increased free iron by +13.26 % (p = 0.019) versus placebo. Furthermore GSE slightly depressed triglyceridemia by -16.44 % (p = 0.067), increased plasma lipase activity by +29.67 % (p = 0.006), but had no effect on cholesterolemia. 

Table 2(Tab. 2) reported the effect of GSE on some haematological parameters of CKD patients. GSE slightly increased white blood cells (WBC) by +23.48 % (p = 0.276) especially the granulocytes. GSE also prevented anemia of CKD patients. A decrease tendency in red blood cells (RBC) from 4.72 ± 0.14 to 4.37 ± 0.19 in control placebo patients (p = 0.127) was noticed which was cleaned by GSE treatment, allowing the stabilization of RBC from 4.22 ± 0.21 ( control 0 month GSE) to 4.23 ± 0.25 (6 months treatment GSE) (p = 0.692). Furthermore GSE did not affect haemoglobin nor did it prevent the decrease in haematocrit of CKD patients from 39.33 ± 0.81 to 35.53 ± 0.96 in control (p = 0.016) and from 37.22 ± 0.84 to 33.30 ± 1.42 in GSE treated patients (p = 0.049). However GSE significantly counteracted thrombocytopenia by +24.63 % (p = 0.030) when compared to placebo which increased platelets by +10.93 % (p = 0.327). 

We reported in Table 3(Tab. 3) the effect of a six-month-long GSE treatment on kidney function biomarker as GFR. The initial number of stage 4 and 3 patients tend to decrease from 6 to 2 and from 11 to 10 patients respectively reaching an overall number of 5 patients who swell the ranks of stage 2, rising the cohort of stage 2 patients from 6 to 11 representing + 83.33 %. No GFR change (no improvement nor worsening) occurred in the placebo group. 

## Discussion

The present work is a preliminary and pilot study dealing with the effect of GSE treatment on CKD patients of various renal failure stages. Data mainly show that even when used at a low dosage, GSE exerts a clear beneficial effect on some renal function biomarkers as well as on the antioxidant status of CKD patients. To our knowledge, our work is the first one to describe such relevant and hopeful clinical trials data for million CKD patients worldwide. 

First of all GSE improves some kidney function parameters as it enhances GFR and clearly lowers proteinuria. This result is of utmost importance as reduction in proteinuria to the lowest achievable level is an important predictor of long term renal protection (de Zeeuw et al., 2004[13]), as it is increasingly recognized that proteinuria may actually be pathological and etiological in CKD progress and not just symptomatic (Roscioni et al., 2014[38]). An association between hyperuricemia and CKD in middle-aged population has also been recently described (Zhu et al., 2014[48]). As in our present case GSE affects both plasma, urine urea and uric acid, its clinical use could be envisaged as an uric acid-lowering therapy substitute to allopurinol.

The beneficial effect of GSE on renal function seems to be mediated, at least partially, by its antioxidant and anti-inflammatory properties. After a six-month-period of treatment, GSE counteracts both plasma lipoperoxidation and carbonylation as well as CRP and LDH, and clearly increases plasma CAT and SOD (Rodrigo and Bosco, 2006[37]). Moreover GSE improves some blood parameters that were shown to be linked to CKD as anemia (Tbahriti et al., 2013[41]), thrombocytopenia (Attman et al., 2011[3]), hypertriglyceridemia (Stegmayr, 2014[40]) and low plasma lipase activity (Dorgalaleh et al., 2013[14]). Importantly no patient drop out of the study nor adverse side effects were noted during the entire clinical trial period. Our data corroborate several previous works in the field demonstrating the heightening of oxidative and inflammatory biomarkers along with a decrease in antioxidant biomarkers in CKD patients. As rightly emphasized (Rodrigo and Bosco, 2006[37]), only few studies dealing with the effect of polyphenols in humans are available, and our contribution is the first to show the effectiveness of GSE to improve renal function in CKD patients from various clinical stages. As a support, dietary supplementation with concentrated red grape juice was shown to exert antioxidant and anti-inflammatory effect in haemodialysis patients (Castilla et al., 2008[7]). Moreover our data are in line with recent animal studies demonstrating the usefulness of grape seed polyphenols in the improvement of renal dysfunction (Bao et al., 2015[5]; Zhang et al., 2014[47]; Gao et al., 2014[19]; Ulusoy et al., 2012[43][44]). In particular the use of high dosage GSE (higher than 500 mg/kg) was recently shown to exert relevant beneficial health effects (Jhun et al., 2013[22]; Tsao et al., 2012[42]; Yang et al., 2012[46]) whereas lower dosages were less effective (Goey et al., 2013[20]).

Remarkably that in our present case, GSE improvement in kidney function of CKD patients has been obtained at a low GSE dosage of 35 mg/kg if we consider the weight of the starting grape seed powder, and highly lower if we consider the polyphenol content. Our laboratory has recently demonstrated the efficiency of high dosage GSE (till 4 g/kg) in multi-organ protection against various insults as obesity (Charradi et al., 2011[11], 2012[10], 2013[9]), stroke (Safwen et al., 2015[39]), or chemotherapeutic treatment (Mokni et al., 2012[32]). In fact, our studies were inspired from the pionneering work of Bagchi et al. (2000[4]) and other subsequent studies that led to the elaboration of calibrated and commercial GSE preparations which evidenced the safety of high dosage GSE (till 2 g/kg) in long-term experiments (Bentivegna and Whitney, 2002[6]). In light of these data it appears that the concentration of GSE used in the present study (35 mg/kg) is far less from the No Observed Adverse Effect Level (NOAEL) of IH 636 or oligonol GSE preparation (Fujii et al., 2007[18]). In addition as GSE recently received the Generally Recognized As Safe (GRAS) certification from US FDA (2011[16]), its use opens a new and safe approach for the validation of high dosage GSE to impede CKD. Most importantly and although used at very low level, GSE seems to slow down the progression of CKD and even to reverse the decline in kidney function (Choi et al., 2014[12]) as described in the present pilot study i.e from stages 4 and 3 to stage 2.

Nevertheless the current interventional pilot study suffers from several limitations. First the sample size is too small to be of robust statistical significance. Although GSE was used at low dosage, a significant improvement in the decline of some kidney function biomarkers after a six-month-treatment period was obtained. Duration of the study should be extended to increase blood and urine sampling and a ten fold higher GSE dosage (350 mg/kg) should be tested to reach improvement of statistical significance. Owing to its tremendous safety, GSE is increasingly used at high dosage (more than 500 mg/kg) according to our own data (Charradi et al., 2011[11], 2014[8], 2012[10], 2013[9]) or to current literature (Li et al., 2015[27]; Mansouri et al., 2011[28]; Margalof et al., 2015[29]).

In conclusion, due to the multi-organ safety and beneficial health effects of grape polyphenols, clinical trials using high GSE dosage should be urgently planned to confirm these preliminary and hopeful data.

## Conflict of interest

The authors declare no conflict of interest.

## Figures and Tables

**Table 1:**
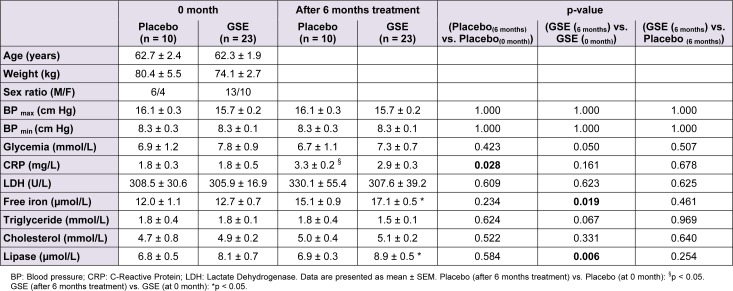
Effect of GSE on anthropometric parameters, plasma biochemistry and inflammation of CKD patients

**Table 2:**
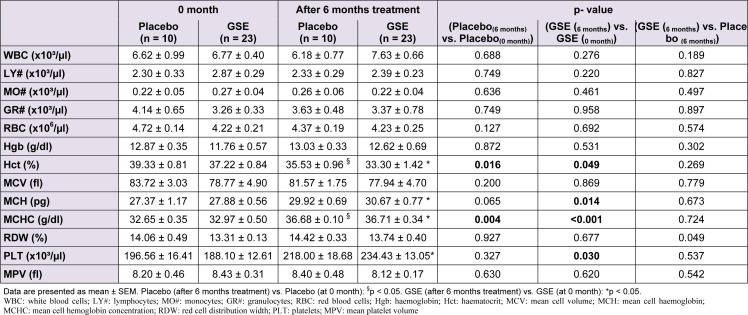
Effect of GSE on CKD patients hematology

**Table 3:**
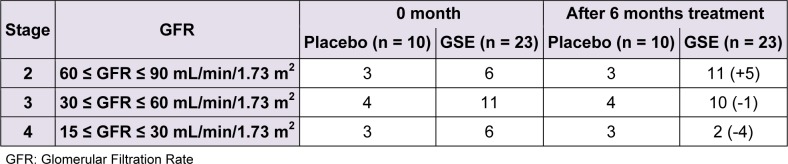
Effect of GSE on the progression of CKD stages
